# Analysis of Public Perception of the Israeli Government’s Early Emergency Instructions Regarding COVID-19: Online Survey Study

**DOI:** 10.2196/19370

**Published:** 2020-05-15

**Authors:** Anat Gesser-Edelsburg, Ricky Cohen, Rana Hijazi, Nour Abed Elhadi Shahbari

**Affiliations:** 1 School of Public Health University of Haifa Haifa Israel; 2 The Health and Risk Communication Research Center University of Haifa Haifa Israel

**Keywords:** covid-19, risk, perception, crisis management, economical threat, compliance to guidelines, spokesperson credibility, source of information credibility, online survey, public health, pandemic

## Abstract

**Background:**

On March 11, 2020, the World Health Organization (WHO) officially declared coronavirus disease (COVID-19) to be a pandemic. This posed challenges to many countries, prominent among which is communication with the public to gain their cooperation. Israel faces different challenges from other countries in its management of the COVID-19 crisis because it is in the midst of a deep constitutional crisis.

**Objective:**

The objective of this paper was to examine the response of the Israeli public to the government’s emergency instructions regarding the pandemic in terms of correlations between overall risk perception and crisis management; overall risk perception and economic threat perception; crisis management and compliance with behavioral guidelines; and crisis management and economic threat perception. We also made comparisons between crisis management and spokesperson credibility and between crisis management and the credibility of information sources.

**Methods:**

The sample was established using an online survey that enabled rapid and effective distribution of an online questionnaire during the COVID-19 crisis. The self-selection online survey method of nonprobability sampling was used to recruit participants (N=1056) through social network posts asking the general public (aged ≥18 years) to answer the survey.

**Results:**

Participants aged ≥65 years perceived higher personal risk compared to those aged 18-30 years (mean difference 0.33, 95% CI 0.04-0.61) and those aged 46-64 years (mean difference 0.38, 95% CI 0.12-0.64). Significant correlations were found between overall risk perception and attitudes toward crisis management (*r*=0.19, *P*<.001), overall risk perception and economic threat perception (*r*=0.22, *P*<.001), attitudes toward crisis management and compliance with behavioral guidelines (*r*=0.15, *P*<.001), and attitudes toward crisis management and economic threat perception (*r*=–0.15, *P*<.001). Participants who perceived that the prime minister was the most credible spokesperson evaluated the crisis management significantly higher than all other groups. The crisis management was evaluated significantly lower by participants who stated that infectious disease specialists were the most credible spokespersons. Participants for whom the Ministry of Health website was the most credible source of information evaluated the crisis management higher than all other groups. Participants for whom scientific articles were the most credible source of information evaluated the crisis management lower than those who perceived that the WHO/Centers for Disease Control and Prevention websites or Ministry of Health/hospital websites and health care workers were the most credible.

**Conclusions:**

The higher the public trust and evaluation of crisis management, the greater the compliance of the public with guidelines. It was also found that crisis management and information cannot be approached in the same way for the overall public. Furthermore, unlike other epidemics, the COVID-19 crisis has widespread economic and social consequences; therefore, it is impossible to focus only on health risks without communicating economic and social risks as well.

## Introduction

On January 30, 2020, the World Health Organization (WHO) declared the coronavirus disease (COVID-19) outbreak to be a Public Health Emergency of International Concern [1]. The virus continued to spread and cross international borders, and on March 11, 2020, the WHO officially declared COVID-19 a pandemic [2,3]. This pandemic has posed challenges to many countries; prominent among these is communicating with the public to gain their cooperation [4,5].

Despite awareness of the centrality and importance of emerging infectious disease communication, many communication failures have occurred globally and locally surrounding successive epidemic outbreaks, such as bovine spongiform encephalopathy (“mad cow disease”), severe acute respiratory syndrome (SARS), H1N1, Zika virus, and Ebola virus [6,7]. Studies have indicated that mistrust in authorities, lack of information transparency, and failure to customize information to different subpopulations are the main reasons for the failures of emerging infectious disease communication over the years [8,9]. Public trust in government institutions and leaders is essential in any country that seeks to impose authority and maintain public order. If citizens of a country do not place trust in authority, the political, economic and social stability of that country are liable to be harmed [10]. Research shows that the degree of trust in the health system has a major impact on public willingness to receive health instructions and to seek out offered services [11,12]. Lack of cooperation and low levels of trust can cause the public to distance itself from the health system, thus exposing individuals and society to health complications [13]. Trusting an institution implies that individuals believe the entity is generally competent, is able to fulfill its obligations toward its constituents, and acts in responsible ways [11]. In decision-making during health crises, individuals must trust the information they receive, and they must trust the organizations and their spokespersons who communicate the information [14,15]. Conveying information and communicating risk to the public during the COVID-19 crisis are becoming complicated issues because of the ongoing uncertainty surrounding the source and spread of the virus and the absence of a vaccine [16-20].

During a health crisis, policymakers must state uncertainty and share all existing information with the public while addressing and customizing the information to different populations to earn public trust [9] and not lose cooperation, such as in the Ebola case in the US [21] and the polio crisis in Israel [22].

On the individual level, the WHO and health authorities have issued instructions to the public on how to avoid contracting COVID-19 [3]. On the state level, COVID-19 has created national emergencies. Each country has established its own policy to manage the epidemic [23,24]. Measures range from strident to lax: China enacted extreme measures, including a general curfew, shutting down air and land travel, prohibiting public gatherings, building hospitals for patients with COVID-19, and hiring health care personnel; South Korea enacted diverse measures, including a combination of monitoring and careful screening based on increasing the number of tests and using electronic surveillance systems to monitor patients [25-27]. Some countries, such as the United States and the United Kingdom, changed their approaches from lenient to stringent in the middle of the global crisis [28].

Israel is one of the countries that responded to the crisis early. On February 27, the first COVID-19 case was confirmed in Israel. After that, schools were shut down, gatherings were prohibited, electronic surveillance measures were introduced by the government to monitor citizens, and emergency regulations were enacted, including imposing a curfew and allowing people to exit their homes only for critical reasons [29].

As of April 12, 2020, the number of confirmed COVID-19 cases in Israel was 10,878, which is an infection rate of 1257 cases per million citizens. There were 103 deaths, which is 12 per million citizens. Relative to 210 countries and territories worldwide, Israel ranked 47th in number of deaths per million citizens and 25th in number of confirmed cases per million citizens. In addition, the number of tests for coronavirus in Israel was 13,577 per million citizens [30].

Israel's management of the COVID-19 crisis differs from that of other countries because Israel is in the midst of a deep constitutional crisis, which is affecting its management of the health crisis. The spread of COVID-19 has created a multidimensional crisis in Israel. Internally, the epidemic is endangering public health, undermining economic and social resilience, challenging effective governance, and even providing cover for processes that could potentially harm democratic values [31].

Currently, the crisis in Israel is being managed by an interim government led by an interim prime minister operating under three criminal indictments after three election cycles in which there was no clear victor. Israel’s parliament had stopped functioning normally [32]. Israeli Prime Minister Benjamin Netanyahu made critical decisions during the COVID-19 crisis, such as shutting down the court system and using surveillance to monitor patients, without bringing his decisions to the cabinet [32]. Some critics argue [33] that some of Prime Minister Netanyahu's decisions were motivated by personal interests (such as closing the courts and thereby postponing his trial) and by political considerations (including the decision to delay the lockdown of centers of contagion, such as ultra-Orthodox communities) [33-35].

In addition to the prime minister, the COVID-19 crisis in Israel is being managed by the director general of the Ministry of Health, who is an economist, and the head of Public Health Services at the Ministry of Health [36]. Most decisions are made by a small team established by Prime Minister Netanyahu [37]. Health Minister Yaakov Litzman (who is not a medical professional) was involved in the decision-making process only at the beginning of the crisis; he refused to take measures against the ultra-Orthodox community, which is his constituency. During the crisis, hospital directors, physicians, and scientists criticized its management [37-39] and called for the replacement of the director general of the Ministry of Health and of Health Minister Yaakov Litzman by health care professionals [40,41]. There was also criticism of the shortages of testing kits [42] and protective equipment for medical workers [43] as well as of how conflicting information was communicated to the public.

In light of the unique confluence in Israel of the health crisis and the constitutional crisis, this study seeks to examine the response of the Israeli public to the Israeli government’s emergency instructions regarding the pandemic in terms of correlations between overall risk perception and crisis management, overall risk perception and economic threat perception, crisis management and compliance with behavioral guidelines, and crisis management and economic threat perception. We also made comparisons between crisis management and spokesperson credibility and between crisis management and the credibility of information sources.

## Methods

### Sampling and Data Collection

The sample was planned using a Qualtrics XM online survey (Qualtrics Survey Software) that enabled rapid and effective distribution of an online questionnaire to our research population. The questionnaire is provided in Multimedia Appendix 1. We used the self-selection online survey method of nonprobability sampling [44] to recruit participants through social network posts asking the general public (people aged ≥18 years) to answer the survey. The rationale for using this sampling method is that the general public in Israel, including the researchers, was under movement restrictions at the time of the study; therefore, distribution of the questionnaire on social networks was more rapid and accessible.

The survey was distributed to the public using three main social media platforms: Facebook, WhatsApp, and Instagram. In the first stage, intensive sampling was accomplished through social networks and social media platforms. In the second stage, snowball sampling [45] was performed to reach broader circles in the Jewish and Arab communities. In the third stage, after a summary meeting and evaluation of the breakdown of the sociodemographic variables, it emerged that the number of participants from the Arab community was higher than that from the Jewish community. Another effort focused on dissemination through diverse circles in the Jewish community, such as community forums, official community Facebook pages, and internal diffusion circles that expanded to broader circles.

A total of 1348 people participated in the survey using the Qualtrics XM platform. However, 292/1348 (27.65%) of the questionnaires were not fully completed or were filled out by participants younger than 18 years. Those questionnaires were taken out of the sample, leaving a total of 1056 eligible participants.

The study was approved by the Faculty of Social Welfare and Health Sciences Ethics Committee for research with human subjects at the University of Haifa (Approval No. 20/088).

### Research Tools

A quantitative questionnaire was designed to test the following variables: risk perception, crisis management, compliance with directives imposed on the public (report of behavioral intentions), and information sources. The questionnaire was based on previous questionnaires culturally accommodated to populations in Israel; it also accommodated the characteristics of the COVID-19 crisis and the measures taken in its wake [46-50].

### Credibility and Validity

Before the questionnaire was distributed, a content validation process was undertaken by performing a pilot study of 20 participants in a limited sampling of the researchers’ narrow circles. The participants were asked to provide feedback on the wording of the questionnaire, the time needed to fill out the questionnaire, etc., and changes were made accordingly. The questions were written in Hebrew and translated into Arabic; subsequently, changes were made in the wording to culturally accommodate it to the specific research population (eg, explanations were provided for certain statements to focus on the participants and prevent information bias).

### Questionnaire Structure and Variable Design

In the first part of the questionnaire (Multimedia Appendix 1), the participants were asked to fill out their demographic information. The second part included an index of questions about risk perception. Some questions focused on the participant’s personal fear of contracting coronavirus, such as “How serious do you perceive COVID-19 to be?” The personal risk perception index was the average of 2 questions (Cronbach α=.76). Other questions focused on fears about different age groups, such as “To what extent do you think the following populations are at high risk of contracting COVID-19?” The overall risk perception index was the average of 17 items (Cronbach α=0.91).

The third part of the questionnaire included an index of 14 questions (statements) on a Likert scale from 1 (not at all) to 5 (very) about the participants’ behavior according to the guidelines given to the Israeli public as a whole. For example: “During the COVID-19 crisis, to what extent do you think you can give up the following behaviors: handshaking, hugging, kissing, not attending social gatherings, etc.?” and “How hard is it for you to follow the guidelines against leaving home to the following destinations?” Compliance to behavioral guidelines was calculated as the average of all 14 items (Cronbach α=.83).

The fourth part was an index of questions about perceptions of the management of the crisis, such as “I think that the measures taken by the state so far to prevent the spread of COVID-19 have been…” or “I think the Prime Minister’s Office and the Ministry of Health are communicating the COVID-19 crisis to the public in a way that is…”

Attitudes on the crisis management index were calculated as the average of 3 questions after recoding the values of each question into 3 levels of evaluation: 1.5 for poor management, 3 for moderately good management, and 4.5 for good management (3 items, Cronbach α=.60).

The fifth part of the questionnaire included a question about economic security: “Beyond the health threat that COVID-19 poses for the public, to what extent does it threaten your economic security?” (1: It is no threat at all to 5: It is a very major threat.)

The sixth part included questions about the credibility of the spokesperson and credibility of the source of information, such as “What do you think is the most credible source of information on COVID-19?” (where participants were asked to mark one information source out of a list of sources) or “Do you feel you are receiving fully transparent information from the Ministry of Health?” (1: Not at all to 5: I receive extremely transparent information.)

### Analysis

A comparison of the personal risk perceptions between age groups was tested using an analysis of variance (ANOVA) model in which the dependent variable was risk perception and the independent variable was age (4 age groups). The specific differences among age groups were tested by post hoc comparisons using the Tukey honestly significant difference (HSD) test.

Correlation between overall risk perception and attitudes toward crisis management and correlation between overall risk perception and economic threat perception were tested using the Pearson correlation coefficient.

Correlation between attitudes toward crisis management and compliance with behavioral guidelines and correlation between attitudes toward crisis management and economic threat perception were tested using the Pearson correlation coefficient.

The relationship between the most credible spokesperson and attitudes toward crisis management was tested using an ANOVA model in which the dependent variable was the attitude toward crisis management and the independent variable was the most credible spokesperson (6 groups, excluding family physicians or other). The specific differences among the 6 groups were tested by post hoc comparison using the Tukey HSD test.

The relationship between the most credible source of information and attitudes toward crisis management was tested using an ANOVA model in which the dependent variable was the attitudes toward crisis management and the independent variable was the source of information (6 sources). The specific differences between credible sources of information and the attitudes toward crisis management among the 6 groups were tested by post hoc comparisons using the Tukey HSD Test.

## Results

### Participants

A total of 1056 eligible participants filled out the online questionnaire (Table 1). 219/1056 (20.74%) were men and 837/1056 (79.26%) were women. The ages of the participants ranged from 18-95 years, with a mean age of 38 years. Of the participants, 423/1050 (40.29%) were Jewish and 627 (59.71%) were Arab. 395/1028 (38.42%) participants were secular, 443 (43.09%) were traditional, and 190 (18.48%) were religious. The participants’ level of education was as follows: 118/1056 (11.17%) secondary education, 102 (9.66%) postsecondary education, 413 (39.11%) BA, 331 (31.34%) MA, 49 (4.64%) PhD, and 43 (4.07%) other types of education.

Because the ethnicity distribution of the sample was not proportional to the general population distribution, a weighting index was calculated. After weighting the data, the ethnicity distribution was 19% Arab and 81% Jewish according to the distribution of people aged ≥18 years in the general population in Israel. To reduce biases, weighting was applied to the data on all statistical inferences (the sociodemographic details presented in Table 1 are based on raw data with no weighting applied).

**Table 1:** Sociodemographic characteristics of the survey participants (N=1056).

Sociodemographic category and characteristics		n (%)
**Gender**		
	Male	219 (20.74)
	Female	837 (79.26)
**Age (years)**		
	18-30	356 (33.78)
	31-45	414 (39.28)
	46-64	224 (21.25)
	>65	60 (5.69)
**Education**		
	Secondary	118 (11.17)
	Postsecondary	102 (9.66)
	BA	413 (39.11)
	MA	331 (31.34)
	PhD	49 (4.64)
	Other	43 (4.07)
**Ethnicity**		
	Jewish	423 (40.29)
	Arab	627 (59.71)
**Religion**		
	Secular	395 (38.42)
	Traditional	443 (43.09)
	Religious	190 (18.48)

### Risk Perceptions

A significant difference in the personal risk perceptions between age groups (*F_3,1050_*=5.14; *P*=.002) was detected. The means and standard deviations of the personal risk indices for the 4 age groups are presented in Table 2.

A significant difference was found between participants aged 65 years and older and participants aged 18-30 years or 46-64 years. Participants aged 65 years and older perceived higher personal risk compared to participants aged 18-30 years (mean difference 0.33, 95% CI 0.04-0.61) and compared to those aged 46-64 years (mean difference 0.38, 95% CI 0.12-0.64). There was no significant difference between participants aged 65 years and older and participants aged 31-45 years in the perception of their personal risk (Table 3).

A significant positive correlation (Pearson) between overall risk perception and attitude toward crisis management was found (r=0.19, *P*<.001). As risk perception increases, the evaluation of the crisis management tends to increase as well.

A significant positive correlation was found between overall risk perception and economic threat perception (r=0.22, *P*<.001). As risk perception increases, the evaluation of the economic threat tends to increase as well, and vice versa; higher economic threat perception is associated with higher risk perception.

**Table 2:** Comparison of the personal risk perceptions between age groups using an ANOVA model (N=1054).

Age group (years)	Personal risk index, mean (SD)
18-30	2.76 (0.55)
31-45	2.84 (0.67)
46-64	2.70 (0.74)
≥65	3.08 (0.94)

**Table 3:** Results of the Tukey HSD test for differences between personal risk perception and age group (N=1054).

Compared age groups (years)	Difference between means (95% CI)
≥65 and 31-45	0.25 (–0.01 to 0.50)
≥65 and 18-30	0.33 (0.04 to 0.61)^a^
≥65 and 46-64	0.38 (0.12 to 0.64)^a^
31-45 and 18-30	0.08 (–0.13 to 0.29)
31-45 and 46-64	0.14 (–0.04 to 0.32)
18-30 and 46-64	0.05 (–0.15 to 0.27)

^a^Statistically significant at α=.05.

### Crisis Management

A significant positive correlation was found between attitudes toward crisis management and compliance with behavioral guidelines (r=0.15, *P*<.001). Higher evaluation of crisis management was associated with higher compliance.

A significant negative correlation was found between attitudes toward crisis management and economic threat perception (r=–0.15, *P*<.001). Higher economic threat perception was associated with lower evaluation of crisis management.

### Spokesperson Credibility

A significant difference (*F_5,981_*=43.16; *P*<.001) between participants who attributed the most credibility to different spokespersons and their attitudes toward crisis management was detected. The means and standard deviations of the attitudes toward crisis management for the six most credible spokespersons (n=987) are presented in Table 4.

Participants for whom the prime minister was the most credible spokesperson evaluated the crisis management significantly higher than all other groups (Table 5). Significantly lower evaluation of the crisis management was expressed by participants for whom infectious disease specialists were the most credible spokespersons compared to those who considered the director general of the Ministry of Health, the head of Public Health Services, or the Minister of Health to be most credible. Participants for whom journalists were the most credible spokespersons evaluated the crisis management significantly lower than those who believed that the director general of the Ministry of Health or the head of Public Health Services was the most credible.

**Table 4:** The relationships between the six most credible spokespersons and the attitudes toward crisis management using an ANOVA model (n=987).

Most credible spokesperson	Mean (SD)
Israeli Prime Minister	3.84 (0.44)
Director general of the Ministry of Health	3.58 (0.49)
Head of Public Health Services	3.49 (0.45)
Israeli Minister of Health	3.33 (0.34)
Infectious disease specialists	2.92 (0.68)
Journalists	2.87 (0.45)

**Table 5:** Results of the Tukey HSD test for differences between the most credible spokesperson and the attitudes toward crisis management (n=987).

Spokesperson comparison			Difference between means (95% CI)
**Prime Minister**			
	Director general of the Ministry of Health	0.26 (0.03 to 0.48)^a^	
	Head of Public Health Services	0.35 (0.09 to 0.61)^a^	
	Minister of Health	0.51 (0.11 to 0.90)^a^	
	Infectious disease specialists	0.92 (0.70 to 1.14)^a^	
	Journalists	0.97 (0.48 to 1.45)^a^	
**Director general of the Ministry of Health**			
	Head of Public Health Services	0.09 (–0.13 to 0.31)	
	Minister of Health	0.25 (–0.12 to 0.62)	
	Infectious disease specialists	0.66 (0.49 to 0.83)^a^	
	Journalists	0.71 (0.24 to 1.19)^a^	
**Head of Public Health Services**			
	Minister of Health	0.15 (–0.24 to 0.55)	
	Infectious disease specialists	0.57 (0.35 to 0.78)^a^	
	Journalists	0.62 (0.13 to 1.11)^a^	
**Minister of Health**			
	Infectious disease specialists	0.41 (0.05 to 0.78)^a^	
	Journalists	0.46 (–0.11 to 1.04)	
**Infectious disease specialists**			
	Journalists	0.05 (–0.42 to 0.52)	

^a^Statistically significant at α=.05.

### Source of Information Credibility

A significant difference (*F_5,1036_*=18.15; *P*<.001) was detected between participants who attributed the most credibility to different information sources and their attitudes toward crisis management. The means and standard deviations of the attitudes toward crisis management for the six most credible information sources are presented in Table 6.

Participants for whom the Ministry of Health website was the most credible source of information evaluated the crisis management higher than all other groups (Table 7).

Participants for whom scientific articles were the most credible source of information evaluated the crisis management lower than those who believed the WHO and Centers for Disease Control and Prevention (CDC) websites or health maintenance organization (HMO)/hospital websites and health care workers were the most credible.

**Table 6:** The relationships between the most credible information sources and the attitudes toward crisis management using an ANOVA model (N=1042).

Most credible information source	Mean (SD)
Ministry of Health website	3.61 (0.46)
WHO^a^/CDC^b^ websites	3.23 (0.61)
HMO^c^/hospital websites and health care workers	3.19 (0.64)
Google/social networks	3.17 (0.68)
Media (television/newspapers)	3.07 (0.58)
Scientific articles	2.87 (0.77)

^a^WHO: World Health Organization.

^b^CDC: Centers for Disease Control and Prevention.

^c^HMO: health maintenance organization.

**Table 7:** Results of the Tukey HSD test for difference between most credible source of information and the attitudes toward crisis management (N=1042).

Source of information		Difference between means (95% CI)
**Ministry of Health website**		
	WHO^a^/CDC^b^ websites	0.38 (0.20-0.56)^c^
	HMO^d^/hospital websites and health care workers	0.42 (0.20-0.65)^c^
	Google/social networks	0.44 (0.02 to 0.87)^c^
	Media (television/newspapers)	0.55 (0.17 to 0.93)^c^
	Scientific articles	0.74 (0.49 to 0.99)^c^
**WHO/CDC websites**		
	HMO/hospital websites and health care workers	0.04 (–0.18 to 0.26)
	Google/social networks	0.06 (–0.37 to 0.49)
	Media (television/newspapers)	0.16 (–0.21 to 0.54)
	Scientific articles	0.36 (0.11 to 0.61)^c^
**HMO/hospital websites and health care workers**		
	Google/social networks	0.02 (–0.42 to 0.47)
	Media (television/newspapers)	0.13 (–0.27 to 0.52)
	Scientific articles	0.32 (0.04 to 0.60)^c^
**Google/social networks**		
	Media (television/newspapers)	0.10 (–0.44 to 0.64)
	Scientific articles	0.29 (–0.17 to 0.76)
**Media (television/newspapers)**		
	Scientific articles	0.19 (–0.23 to 0.61)

^a^WHO: World Health Organization.

^b^CDC: Centers for Disease Control and Prevention.

^c^Statistically significant at α=.05.

^d^HMO: health maintenance organization.

## Discussion

### Principal Findings

This study was conducted during March 2020 in Israel. We sought to examine public perceptions of risk concerning COVID-19 and public assessment of policymakers’ management of the crisis. The findings indicate that participants aged 65 years and older have a higher risk perception of contracting COVID-19 than the younger age groups. This finding is consistent with scientific facts indicating that older people are at highest risk due to the severity of the illness and the fatality rate [51]. The older age group indicated that its fears are science-based and were not false concerns. Similarly, the younger age groups were less afraid, which is consistent with their relative risk.

The findings of this study also indicate that the greater the participant’s personal risk perception, the better they evaluated the crisis management, and vice versa. A possible explanation of this finding is that people who are very concerned about COVID-19 are at such a high level of fear that they view any action taken by policymakers to confront and combat the virus as reasonable. Furthermore, the actions taken by Israel, such as requiring people returning from abroad to enter quarantine since the beginning of the crisis before there were any fatalities in Israel; grounding of flights; cancellation of public events and gatherings; surveillance and phone tracking of patients; and curfew on the entire public were perceived by people who are very afraid of the disease as appropriate and not excessive. To reinforce this interpretation, it was found in studies that higher levels of perceived susceptibility are associated with greater intention to change behavior in the manner recommended in the fear appeal message; also, a higher level of perceived susceptibility is a strong determinant of intentions and behavior, even in the face of weak arguments [52]. On the other hand, people with lower perceived susceptibility evaluated the crisis management as less good, possibly for the opposite reason: they view the draconian measures taken by Israel, including an arbitrary curfew on the entire population (including subpopulations that were not at risk) and a curfew on geographical areas where contagion was low, as excessive and disproportionate. These measures have drawn public criticism [38,53].

Another finding of the study is a positive significant correlation between overall risk perception and economic threat perception. As risk perceptions increase, the evaluation of the economic threat also tends to increase, and vice versa. This finding indicates that the health crisis caused by the COVID-19 pandemic had far-reaching consequences for the global, national and personal economy; therefore, the participants were afraid not only of the health threat but also the inherent economic threat. It was also found that higher economic threat perception was associated with lower evaluation of crisis management.

It is likely that people who perceive a high personal economic threat feel that the government is not managing the crisis well if it is allowing their economic resilience to be harmed. The feeling that the crisis management is causing fatal harm to the Israeli economy also arises from conversations on social networks and the Israeli media, where it has been argued that the high economic and political price that Israel is paying is even more dangerous than COVID-19 [54].

Another key finding from this study was a positive significant correlation between attitudes toward crisis management and compliance with behavioral guidelines. Studies indicate that public trust in government institutions and leaders is considered essential in any country that seeks to impose its authority on the public and maintain order. High evaluation and trust of the functioning of authorities affects the behavior of the public [11,12]. In the context of this study, the participants’ high evaluation of the crisis management in Israel affected the public’s high compliance with the guidelines during the pandemic. This finding reinforces the importance of trust in the health care system, especially during a crisis such as the COVID-19 pandemic, when the public is asked to change its routine behaviors and habits.

Furthermore, the findings of this study indicate that participants for whom the prime minister was the most credible spokesperson evaluated the crisis management as significantly better than all other groups. The crisis management was evaluated as significantly worse by participants who perceived infectious disease specialists to be the most credible spokespersons compared to those who perceived the most credible spokespersons to be the director general of the Ministry of Health, the head of Public Health Services, or the Minister of Health. These findings indicate the importance of spokespersons during epidemic crises [55].

Selecting appropriate spokespersons to communicate with the public during and after a health crisis is a strategic decision that can have far-reaching results [56-58]. The spokesperson is perceived as the representative of the establishment managing the crisis [55,59,60]. The higher the credibility of the spokesperson, the greater the chance the audience will be open to receiving the messages and complying with the guidelines.

Participants who viewed Prime Minister Netanyahu as the most credible spokesperson evaluated the crisis management as good because he performs two functions: manager and spokesperson of the crisis. Throughout the crisis, Prime Minister Netanyahu appeared at dozens of press conferences and delivered the guidelines to the public himself. Conversely, participants who viewed infectious disease specialists as the most credible spokespersons provided a lower evaluation of the crisis management. The apparent reason is that in Israel, the crisis was managed over the entire period since the COVID-19 crisis began by a very narrow and centralized team [37]. This team was harshly criticized by certain elements in the health care system and the general public. The criticism focused on the insufficient number of public health and medical experts on the team. Due to this criticism, during the crisis, hospital directors and physicians called for the director general of the Ministry of Health, who is an economist, and the health minister, who is not a health professional, to be dismissed and replaced with professionals [40,61]. In epidemic/pandemic crises in the age of new media, it is important for both spokespersons and information sources to be perceived by the public as credible [62-65]. The findings of this study indicate that those who perceived the health ministry to be the most credible information source also perceived the crisis management to be the most favorable, contrary to those who perceived academic articles to be the most credible information sources and perceived the crisis management as less favorable.

It is likely that the participants who perceived the Ministry of Health website (ie, the website that represents the body managing the crisis) as most credible will also perceive the crisis management to be good. Thus, they are exposed to information on the Ministry of Health website, which supports its management decisions with findings and testimonies; this exposure apparently affects their view of the crisis management as optimal. Conversely, people who read academic articles and are not only exposed to informative materials provided to them by the Ministry of Health are likely to be highly literate; therefore, it is likely that they are exposed to other materials and findings that are not consistent with the Ministry of Health guidelines.

Experts have argued that the Israeli Ministry of Health guidelines are contradictory. At the beginning of the COVID-19 crisis, the Israeli Ministry of Health claimed that there was no need for masks, although they were already being used in other countries according to recommendations and prior knowledge [66]; however, later, the guideline was changed to require the public to use masks. In another example of conflicting information, due to the shortage of personal protective equipment (PPE) for its employees, the Ministry of Health initially issued a statement in mid-March stating that health care workers do not need to wear PPE regularly but only in certain situations [67]. Following the Ministry of Health statement, senior physicians from across the country claimed that the Ministry of Health statement was an excuse to cover up the inadequacy of the Israeli health care system. According to the last State Comptroller and Ombudsman of Israel report [68], the PPE shortage is only one example of this inadequacy.

Also, it was found in the literature that vaccine-hesitant groups who show skepticism toward the establishment are exposed to academic articles and do not rely only on government information [49]. Follow-up studies can examine the association between the phenomenon of hesitancy and how hesitant groups perceive the management of the COVID-19 crisis.

### Limitations

The limitations of this study are that it is not a representative study. This study used nonprobability sampling procedures and measuring. Despite the nonprobability sampling, the sample included a high total number of participants. Secondly, since the research was conducted during the COVID-19 crisis and it was important to examine the public's positions regarding crisis management, we decided to distribute the survey online on social networks to reach a broad circle of people in a short time. Furthermore, during the COVID-19 crisis, the public was required to maintain social distancing; therefore, an online survey was the most suitable tool. However, the sociodemograpic statistics presented suggest that a diverse sample was reached based on sociodemographic variables. Since the ethnicity distribution of the sample was not proportional to the general population distribution, a weighting index was calculated. The ethnicity distribution after weighting the data was 19% Arab and 81% Jewish according to the distribution of the general population aged ≥18 years in Israel. To reduce biases, weighting was applied to the data on all statistical inferences.

### Conclusions

This study suggests that it is critical to establish public trust in decision makers. The higher the public trust and evaluation of crisis management, the more the public will comply with guidelines. It was also found that the crisis management and information cannot be approached in the same way for the overall public. Decision makers must address and communicate the risks differently to different subpopulations that have different risk perceptions and different levels of health literacy. Furthermore, unlike other epidemic crises, the COVID-19 crisis has widespread economic and social consequences; therefore, it is impossible to communicate and focus only on the health risk without communicating the economic and social risks as well.

## Appendix

Multimedia Appendix 1 The online survey questionnaire.

## Abbreviations

ANOVA: analysis of variance
CDC: Centers for Disease Control and Prevention
COVID-19: coronavirus disease
HMO: Health Maintenance Organization
HSD: honestly significant difference
PPE: personal protective equipment
SARS: severe acute respiratory syndrome
WHO: World Health Organization
