# Supplementary material for: Analysis of Public Perception of the Israeli Government’s Early Emergency Instructions Regarding COVID-19: Online Survey Study
Source: J Med Internet Res. 2020 May 15;22(5):e19370. doi: 10.2196/19370 (PMC7236609; doi:10.2196/19370)
Supplement: Multimedia Appendix 1 [file jmir_v22i5e19370_app1.docx]

**Multimedia Appendix 1:** The online survey questionnaire

Due to the COVID-19 (coronavirus) crisis currently afflicting the whole world including Israel, we would like to learn from you how you are coping and how you perceive the crisis management in Israel. 
This questionnaire is anonymous. There are no right or wrong answers. If you feel uncomfortable you may stop answering the survey at any time. The questionnaire is short (5-10 minutes) and friendly and the information provided will serve exclusively for the purposes of the study.
Your participation is important to us and we thank you in advance for your help.
Sincerely,
Health and Risk Communication Research Center, Haifa University

If you are over 18 years old and agree to answer the survey, press the arrow to continue.

1. Your sex: 1. Male 2. Female

2. Age:_____

3. Do you have children? 1. Yes 2. No

4. Education: 1. Secondary 2. Post-secondary 3. BA 4. MA 5. PhD 6. Other________

5. Profession: _____________

6. Geographical place of residence: _____________

7. Ethnicity: 1. Jewish 2. Arab 3. Druze 4. Circassian 5. Other__________

8. Religion: 1. Jewish 2. Muslim 3. Christian 4. Druze 5. Circassian 6. Other__________

9. Religiosity: 1. Secular 2. Traditional 3. Religious 4. Haredi 5. Other_________

10. To what extent if at all do you feel you are at risk of contracting the COVID-19? 1. No risk at all 2. Low risk 3. Medium risk 4. High risk 5. Very high risk

11. Please rate the following statements from 1 (not afraid at all) to 5 (very afraid).

|  | 1. Not afraid at all | 2. Somewhat afraid | 3. Moderately afraid | 4. Quite afraid | 5. Very afraid |
| --- | --- | --- | --- | --- | --- |
| 1. I am personally afraid of contracting the COVID-19. |  |  |  |  |  |
| 2. I'm afraid of my children contracting the COVID-19. |  |  |  |  |  |
| 3. I'm afraid of my family members (spouse, parents, brothers and sisters) contracting the COVID-19. |  |  |  |  |  |
| 4. I'm afraid of people in my immediate surroundings (friends, neighbors, work colleagues) contracting the COVID-19. |  |  |  |  |  |
| 5. I'm afraid most of the community in which I live will contract the COVID-19. |  |  |  |  |  |

12. How serious do you perceive COVID-19 to be? 1. No risk at all 2. Low risk 3. Moderate risk 4. High risk 5. Very high risk.

13. To what extent do you think the following populations are at high risk of contracting the COVID-19?

|  | 1. No risk at all | 2. Low risk | 3. Moderate risk | 4. High risk | 5. Very high risk |
| --- | --- | --- | --- | --- | --- |
| 1. Babies ages 0-2 |  |  |  |  |  |
| 2. Children up to age 18 |  |  |  |  |  |
| 3. The young adult population (19-65) |  |  |  |  |  |
| 4. The population with comorbidity (such as heart disease, diabetes, hypertension) regardless of age |  |  |  |  |  |
| 5. The elderly population (65+) |  |  |  |  |  |

14. To what extent if at all do you think contagion of the following population would risk their lives?

|  | 1. No risk at all | 2. Low risk | 3. Moderate risk | 4. High risk | 5. Very high risk |
| --- | --- | --- | --- | --- | --- |
| 1. Babies ages 0-2 |  |  |  |  |  |
| 2. Children up to age 18 |  |  |  |  |  |
| 3. The young adult population (19-65) |  |  |  |  |  |
| 4. The population with comorbidity (such as heart disease, diabetes, hypertension) regardless of age |  |  |  |  |  |
| 5. The elderly population (65+) |  |  |  |  |  |

15. Do you think the COVID-19 crisis will go away in the summer? 1. Yes 2. No

16. What do you think is the source of the COVID-19? 1. The virus moved from bats to human beings at a food market in Wuhan, China 2, The virus was always present but underwent a mutation 3. The virus was accidentally leaked from a bioterrorism laboratory in Wuhan, China 4. An attempt to cull the world’s population 5. US biological warfare in China 6. A Chinese conspiracy to rule the world economy 7. I don't know 8. Other________

17. How is the COVID-19 transferred from one person to another? (You can mark more than one answer). 1. Through air – cough, sneeze, or speech 2. Contact with body fluids – eye mucus and nose and mouth discharges 3. Contact with surfaces containing droplets with the virus 4. Through blood 5. I don't know 6. Other____________

18. How much do you think the health organizations are responding (with reasoned answers) to various theories spread online about the source of the COVID-19? 1. Provide reasoned answers to each theory 2. Define them as misinformation but do not explain why 3. Ignore them and do not address the various theories 4. Answer partially 5. I don't know 6. Other________________

19. Do you feel you are receiving fully transparent information from the MOH? 1. Not at all 2. I receive minimally transparent information 3. I receive moderately transparent information 4. I receive very transparent information 5. I receive extremely transparent information

20. Which information source would you go to for information about the COVID-19? Please mark the main two sources you would turn to. 1. A healthcare worker (doctor/nurse/public health expert) 2. The World Health Organization (WHO) and Centers for Disease Control (CDC) websites 3. Ministry of Health website 4. Social network and forums (Facebook, Twitter, blogs) 5. Scientifc articles 6. Google 7. Friends 8. Work colleagues 9. Family 10. Television 11. Newspapers 12. HMO and hospital websites 13. Clergy

21. What do you think is the most credible source of information on the COVID-19? (Please mark a single answer) 1. A healthcare worker (doctor/nurse/public health expert) 2. The World Health Organization (WHO) and Centers for Disease Control (CDC) websites 3. Ministry of Health website 4. Social network and forums (Facebook, Twitter, blogs) 5. Scientifc articles 6. Google 7. Friends 8. Work colleagues 9. Family 10. Television 11. Newspapers 12. HMO and hospital websites 13. Clergy

22. Who do you think is the most credible spokesperson during the COVID-19 crisis in Israel? 1. The Prime Minister 2. The Director General of the Ministry of Health 3. The Health Minister 4. The head of the public health services at the Ministry of Health 5. Journalists 6. Infectious disease experts 7. Family physicians 8. Other__________________________

23. What do you feel you are not getting from the authorities during the COVID-19 crisis? (information, economic support, psychological support etc.') _____________

24. I think that the measures taken by the state so far to prevent the spread of the covid-19 have been: 1. Vital 2. Excessive 3. Unnecessary 4. I don’t know 5. Other__________________________

25. I feel that the measures taken by the state so far to prevent the spread of the COVID-19: 1. Reassure me because they protect my and my family’s health 2. Unsettle me because I am uncertain and don't know what is going to happen 3. Anger me because I am paying a heavy price for them 4. Give me mixed feelings (both reassuring me and unsettling me) 5. I don’t know 6. Other ______________________________________________________

26. I think the Prime Minister’s Office and the Ministry of Health are communicating the COVID-19 crisis to the public in a way that is 1. Clear and transparent 2. Clear but sometimes partial 3. Contradictory (saying one thing and then the opposite) 4. Other____________________

27. The strategy used by the Prime Minister's office and health ministry to communicate the COVID-19 crisis is: 1. A strategy of intimidation 2. A strategy of empathy 3. A strategy of full transparency 4. A strategy of encouraging social solidarity 5. I don't know 6. Other ______________________________________________________

28. During the COVID-19 crisis, to what extent do you think you can give up the following behaviors: on a scale from 1 to 5 (1 - very easy to give up, 5 - very hard to give up)

|  | 1. Very easy to give up | 2. Easy to give up | 3. Moderately easy to give up | 4. Fairly hard to give up | 5. Very hard to give up |
| --- | --- | --- | --- | --- | --- |
| 1. Handshaking |  |  |  |  |  |
| 2. Hugging |  |  |  |  |  |
| 3. Kissing |  |  |  |  |  |
| 4. Keeping a 2 m distance between people |  |  |  |  |  |
| 5. Not attending social encounters of more than 10 people |  |  |  |  |  |

29. How hard is it for you to follow the guideline against leaving home to the following destinations? On a scale of 1 to 5 (1 - not hard at all, 5- very hard)

|  | 1 . Not hard at all | 2 . Somewhat hard | 3 . Moderately hard | 4 . Very hard | 5 . Extremely hard |
| --- | --- | --- | --- | --- | --- |
| 1. Attending a wedding |  |  |  |  |  |
| 2. Making a condolence call |  |  |  |  |  |
| 3. Praying at a praying site |  |  |  |  |  |
| 4. Going to the supermarket or shopping center |  |  |  |  |  |
| 5. Going to work |  |  |  |  |  |
| 6. Taking public transportation |  |  |  |  |  |
| 7. Going to the doctor for a checkup |  |  |  |  |  |
| 8. Visiting a relative in the hospital |  |  |  |  |  |
| 9. A social gathering of more than 10 people |  |  |  |  |  |

30. In light of the new guidelines closing down schools, is it possible to prevent a family member who is supposed to be in quarantine from being in a shared space with other family members? 1. Yes it works 2. It works only partially 3. It doesn't work 4. I don’t know

31. Beyond the health threat the COVID-19 poses for the public, to what extent does it threaten your economic security? 1. It is no threat at all 2. It is a small threat 3. It is a moderate threat 4. It is a major threat 5. It is a very major threat

32. In addition to the guidelines given to the public, do you take your own measures to protect yourself and your environment from contagion? If so, please specify what you do? _____________
